# Co-Producing and Quality Assuring Multi-Modal Psychoeducation to Enable Early Engagement in Guided Self-Help for People With Functional Neurological Disorder

**DOI:** 10.1192/bjo.2024.357

**Published:** 2024-08-01

**Authors:** Tahani Dahir, Stephanie Lapitan, Adna Mohamud, Anushka Saxena, Verity Williams

**Affiliations:** 1 King's College London, London, United Kingdom; 2 Kent & Medway Partnership Trust, Kent, United Kingdom

## Abstract

**Aims:**

People with Functional Neurological Disorder (FND) exhibit diverse symptoms, ranging from motor and sensory issues to non-epileptic attacks, potentially causing reduced functioning and quality of life. East Kent Neuropsychiatry Service developed written and video resources to educate patients about FND. We aim to improve patient education on FND through increasing resource options and identifying optimal implementation of the materials within the care pathway.

**Methods:**

We implemented an existing symptom self-management psychoeducation booklet and novel video resources as part of a quality improvement project (QIP). The first QIP cycle trialled the resources across different treatment pathways using three groups, each of seven patients. Group 1 received the booklet first, then the video two weeks later. Group 2 received the video first, then the booklet after two weeks. Group 3 received both resources at the same time. After 4 weeks, patient feedback was collected by 4 medical students by telephone. Qualitative and quantitative data was obtained from 8 patients. Quantitative feedback was obtained using a 5-point Likert scale. In the second QIP cycle, 10 patients received both resources simultaneously, with improvements made to resource accessibility and readability.

**Results:**

The first QIP cycle highlighted that the videos were helpful in explaining FND, with 75% of patients rating the videos the same or higher than the booklet. Qualitative responses commented that videos were more personal and easier for family members to understand. Across both video and booklet resources, 67% of patients agreed or strongly agreed the resources were useful for explaining FND and their experience. One patient, in group 1, stated the resources improved their symptoms. 54% of patients agreed that they received the resources at the appropriate time; a common theme across all groups was the desire to access the resources earlier within the pathway. In the second QIP cycle (8 patients, 25% response rate), all agreed the resources improved FND understanding and self-management strategies.

**Conclusion:**

Our study highlights that video resources are a valuable addition to FND psychoeducation, with benefits for patients, carers and family members. Both booklet and video resources were helpful in improving patient education on FND. Our findings emphasise the need for early integration of psychoeducation in the care pathway. Future developments could include collaborating with other specialties involved in the care of FND patients, such as neurology and emergency departments, to enable early integration of psychoeducation resources, empowering clinicians to effectively communicate about FND and enhancing patient psychoeducation.

Additional authors: Mr Alan Dunlop, Ms Wendy Collison and Professor Rafey Faruqui.

